# Supplementary figures and images for: Structure-Guided Design of a Synthetic Mimic of an Endothelial Protein C Receptor-Binding PfEMP1 Protein
Source: mSphere. 2021 Jan 6;6(1):e01081-20. doi: 10.1128/mSphere.01081-20 (PMC7845591; doi:10.1128/mSphere.01081-20)

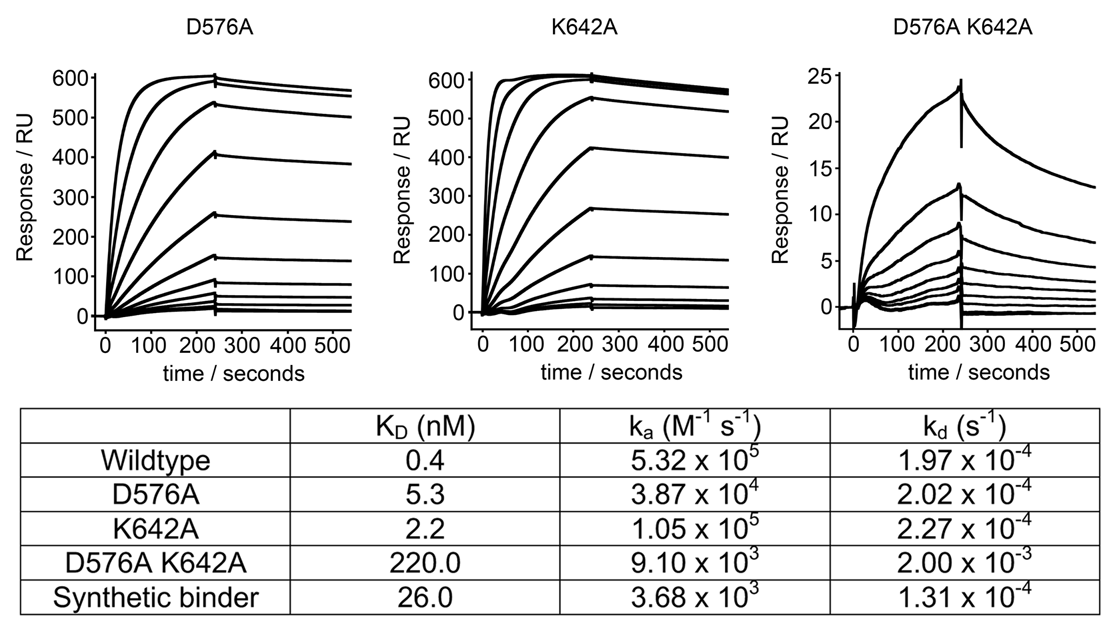

Supplement: FIG S1 [file mSphere.01081-20-sf001.tif]

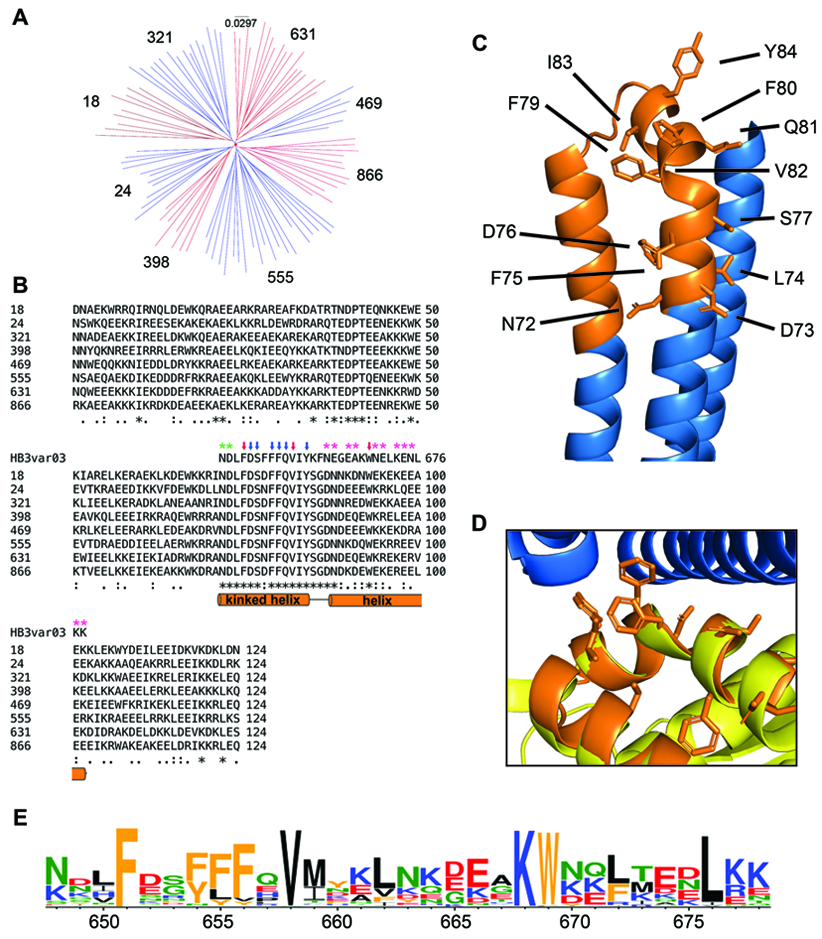

Supplement: FIG S2 [file mSphere.01081-20-sf002.tif]

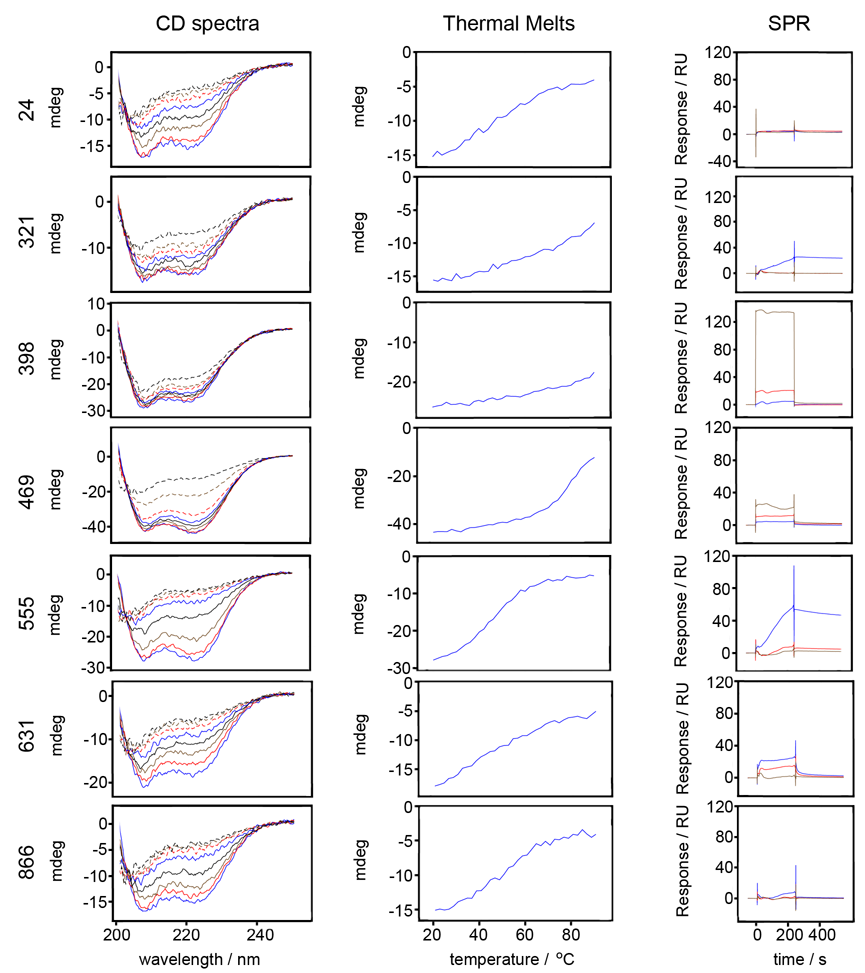

Supplement: FIG S3 [file mSphere.01081-20-sf003.tif]

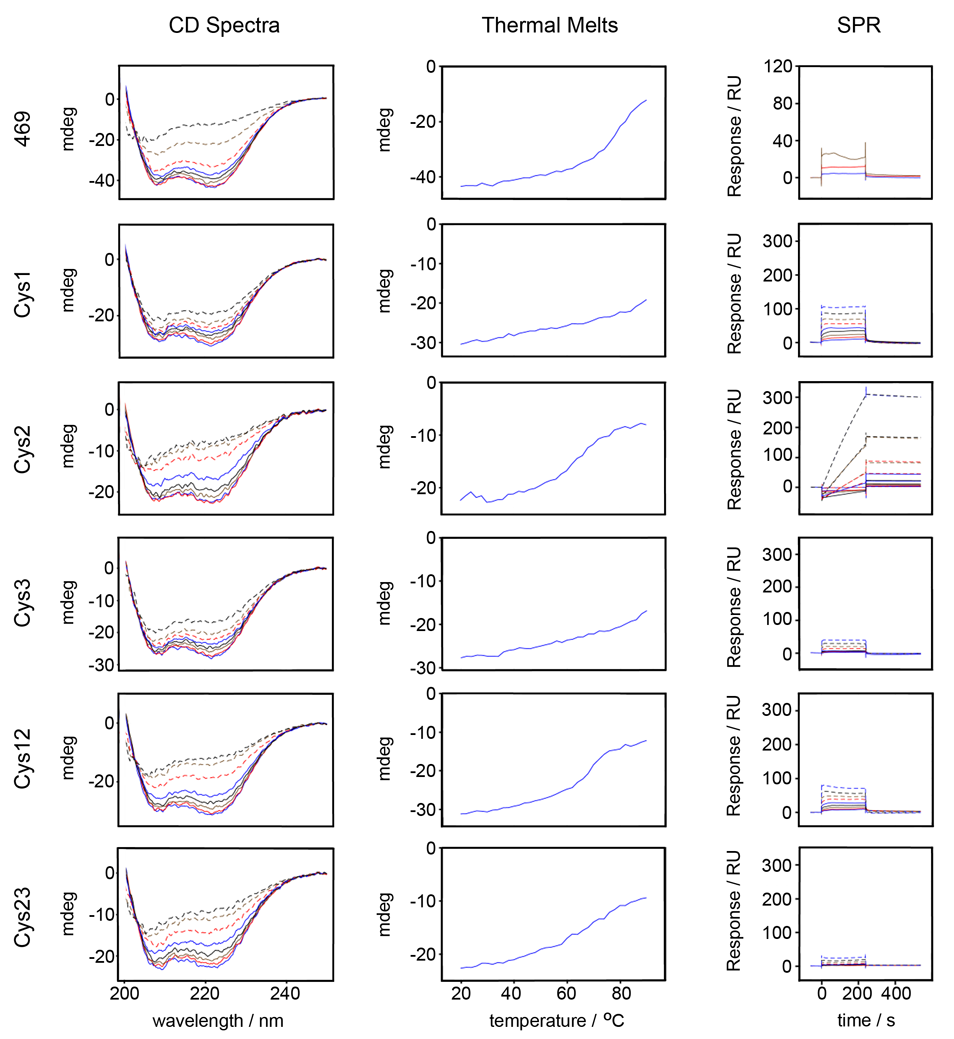

Supplement: FIG S4 [file mSphere.01081-20-sf004.tif]

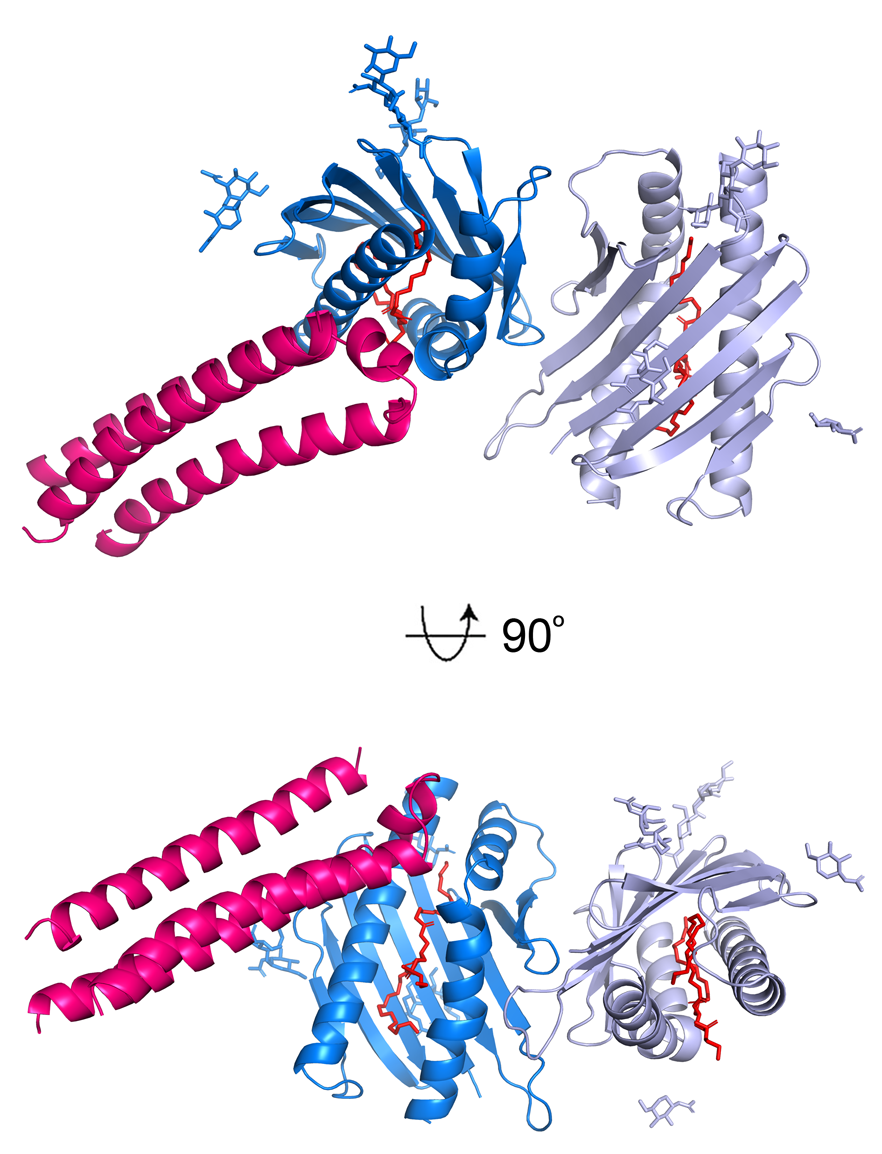

Supplement: FIG S5 [file mSphere.01081-20-sf005.tif]

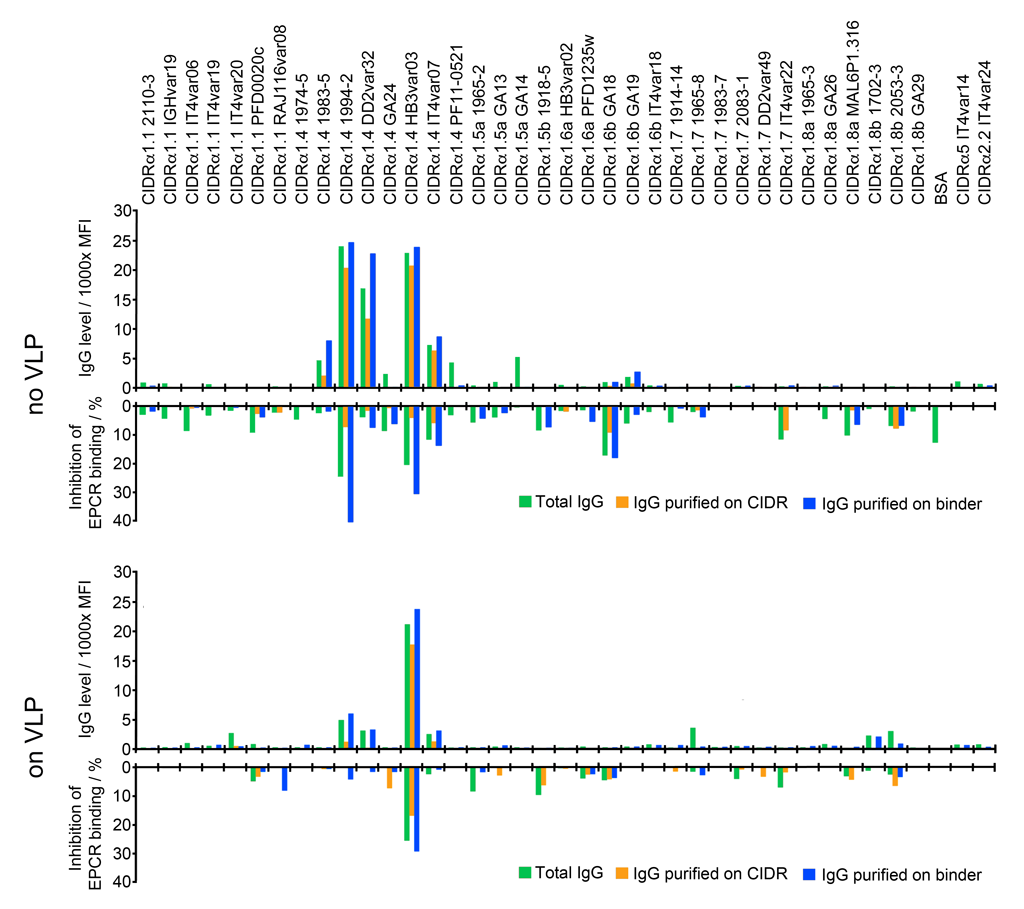

Supplement: FIG S6 [file mSphere.01081-20-sf006.tif]
