# Supplementary material for: Structure-Guided Design of a Synthetic Mimic of an Endothelial Protein C Receptor-Binding PfEMP1 Protein
Source: mSphere. 2021 Jan 6;6(1):e01081-20. doi: 10.1128/mSphere.01081-20 (PMC7845591; doi:10.1128/mSphere.01081-20)
Supplement: TABLE S1 [file mSphere.01081-20-st001.docx]

***Table S1: Sequences of synthetic binders:***

***0018:***

***DNA:***

GATAATGCAGAAAAATGGCGTCGTCAGATTCGTAATCAGCTGGATGAATGGAAACAGCGTGCCGAAGAAGCACGTAAACGCGCACGTGAAGCATTTAAAGATGCAACCCGTACCAATGATCCGACCGAACAGAACAAAAAAGAATGGGAAAAAATTGCCCGTGAGCTGAAAGAACGTGCCGAAAAACTGAAAGATGAGTGGAAAAAACGCATCAACGACCTGTTTGATAGCGATTTTTTTCAGGTGATCTATAGCGGTGACAACAACAAAGATAACTGGGAGAAAGAAAAAGAAGAAGCCGAAAAAAAACTGGAAAAATGGTATGACGAAATCCTGGAAGAGATCGACAAAGTGAAAGATAAACTGGATAATTAA

***Protein:***

DNAEKWRRQIRNQLDEWKQRAEEARKRAREAFKDATRTNDPTEQNKKEWEKIARELKERAEKLKDEWKKRINDLFDSDFFQVIYSGDNNKDNWEKEKEEAEKKLEKWYDEILEEIDKVKDKLDN

***0024:***

***DNA:***

AATAGCTGGAAACAAGAAGAAAAACGTATTCGCGAAGAAAGCGAAAAAGCCAAAGAAAAAGCCGAAAAACTGAAAAAACGTCTGGATGAATGGCGTGATCGTGCACGTCAGACCGAAGATCCGACCGAAGAAAATGAGAAAAAATGGAAAGAAGTGACCAAACGCGCAGAAGAGGATATCAAAAAAGTGTTCGATGAGTGGAAAGATCTGCTGAATGACCTGTTTGATAGCAACTTTTTCCAGGTGATTTATAGCGGTGATAACGATGAAGAAGAGTGGAAACGTAAACTGCAAGAAGAGGAAGAGAAAGCCAAAAAAGCAGCACAAGAAGCCAAACGTCGTCTGGAAGAAATCAAAAAAGATCTGCGCAAATAA

***Protein:***

NSWKQEEKRIREESEKAKEKAEKLKKRLDEWRDRARQTEDPTEENEKKWKEVTKRAEEDIKKVFDEWKDLLNDLFDSNFFQVIYSGDNDEEEWKRKLQEEEEKAKKAAQEAKRRLEEIKKDLRK

***0321:***

***DNA:***

AATAATGCAGATGAGGCCGAGAAAAAAATCCGTGAAGAACTGGATAAATGGAAACAAGAAGCAGAACGCGCAAAAGAAGAGGCCGAAAAAGCACGTGAAAAAGCCCGTCAGACCGAAGATCCGACCGAAGAGGCTAAAAAAAAATGGGAAAAACTGATCGAAGAACTGAAAGAGCGTGCAGATAAACTGGCAAATGAAGCAGCAAATCGTATCAACGACCTGTTTGATAGCGATTTTTTCCAGGTGATTTATAGCGGTGATAACAATCGTGAAGATTGGGAAAAAAAAGCCGAGGAAGCAAAAGACAAACTGAAAAAATGGGCTGAAGAAATCAAACGTGAACTGGAACGCATCAAAAAAGAGCTGGAACAGTAG

***Protein:***

NNADEAEKKIREELDKWKQEAERAKEEAEKAREKARQTEDPTEEAKKKWEKLIEELKERADKLANEAANRINDLFDSDFFQVIYSGDNNREDWEKKAEEAKDKLKKWAEEIKRELERIKKELEQ

***0398:***

***DNA:***

AACAACTATCAGAAAAACCGTGAAGAAATTCGTCGTCGTCTGGAACGTTGGAAACGTGAAGCAGAAGAACTGAAACAGAAAATCGAGGAACAGTACAAAAAAGCCACCAAAACCAATGATCCGACCGAGGAAGAGAAAAAAAAATGGGAAGAAGCCGTTAAACAGCTGGAAGAAGAAATCCGTAAACGCGCACAAGAATGGCGTCGTCGTGCAAATGACCTGTTTGATAGCAACTTTTTCCAGGTGATTTATAGCGGTGATAACGATGAACAAGAGTGGAAACGCGAACTGGAAGAGGCAAAAGAGGAACTGAAAAAAGCAGCCGAAGAACTGGAACGTAAACTGGAAGAAGCCAAAAAAAAACTGAAACAATAA

***Protein:***

NNYQKNREEIRRRLERWKREAEELKQKIEEQYKKATKTNDPTEEEKKKWEEAVKQLEEEIRKRAQEWRRRANDLFDSNFFQVIYSGDNDEQEWKRELEEAKEELKKAAEELERKLEEAKKKLKQ

***0469:***

***DNA:***

AATAATTGGGAGCAGCAGAAAAAAAACATCGAGGATGATCTGGACCGTTACAAAAAACGTGCAGAAGAACTGCGTAAAGAAGCCGAAAAAGCACGCAAAGAAGCACGTAAAACCGAAGATCCGACCGAAGAGGCCAAAAAAGAATGGGAAAAACGTCTGAAAGAACTGGAAGAACGTGCCCGTAAACTGGAAGATGAAGCAAAAGATCGTGTGAACGACCTGTTTGATAGCAACTTTTTTCAGGTGATCTATAGCGGTGATAACGATGAAGAAGAGTGGAAAAAAGAAAAAGACCGTGCCGAGAAAGAAATCGAAGAATGGTTTAAACGCATCAAAGAAAAACTGGAAGAGATCAAAAAACGCCTGGAACAGTAA

***Protein:***

NNWEQQKKNIEDDLDRYKKRAEELRKEAEKARKEARKTEDPTEEAKKEWEKRLKELEERARKLEDEAKDRVNDLFDSNFFQVIYSGDNDEEEWKKEKDRAEKEIEEWFKRIKEKLEEIKKRLEQ

***0555:***

***DNA:***

AATAGCGCAGAACAGGCAGAAAAAGATATCAAAGAAGATGACGATCGTTTTCGCAAACGTGCAGAAGAAGCAAAACAGAAACTGGAAGAATGGTATAAACGTGCCCGTCAGACCGAAGATCCGACCCAAGAAAATGAAGAAAAATGGAAAGAAGTTACCGATCGCGCAGAAGATGATATTGAAGAACTGGCAGAACGTTGGAAACGTCGTGCAAATGACCTGTTTGATAGCAACTTTTTCCAGGTGATTTATAGCGGTGACAACAACAAAGATCAGTGGGAAAAACGTCGCGAAGAAGTTGAACGTAAAATCAAACGTGCCGAGGAAGAACTGCGTCGTAAACTGGAAGAGATTAAACGTCGTCTGAAAAGCTAAG

***Protein:***

NSAEQAEKDIKEDDDRFRKRAEEAKQKLEEWYKRARQTEDPTQENEEKWKEVTDRAEDDIEELAERWKRRANDLFDSNFFQVIYSGDNNKDQWEKRREEVERKIKRAEEELRRKLEEIKRRLKS

***0631:***

***DNA:***

AATCAGTGGGAAGAAGAGAAAAAAAAAATCGAAAAAGACGACGATGAGTTCCGTAAACGTGCAGAAGAGGCCAAAAAAAAAGCAGATGATGCATACAAAAAAGCCCGTAAAACCGAAGATCCGACCGAGGAAAACAAAAAACGTTGGGATGAATGGATCGAAGAACTGAAAAAAGAGATCGAGAAAATTGCCGATCGTTGGAAAGATCGTGCAAATGACCTGTTTGATAGCAACTTTTTCCAGGTGATTTATAGCGGTGATAACGATGAACAAGAATGGAAACGTGAAAAAGAGCGCGTTGAGAAAGATATTGATCGTGCCAAAGATGAACTGGATAAAAAACTGGATGAAGTGAAAGACAAACTGGAAAGCTAA

***Protein:***

NQWEEEKKKIEKDDDEFRKRAEEAKKKADDAYKKARKTEDPTEENKKRWDEWIEELKKEIEKIADRWKDRANDLFDSNFFQVIYSGDNDEQEWKREKERVEKDIDRAKDELDKKLDEVKDKLES

***0866:***

***DNA:***

CGTAAAGCAGAAGAGGCCAAAAAAAAAATCAAACGCGATAAAGATGAAGCCGAAGAGAAAGCCGAAAAACTGAAAGAACGTGCCCGTGAAGCATACAAAAAAGCACGTAAAACCGAAGATCCGACCGAAGAAAATCGTGAAAAATGGGAAAAAACCGTGGAAGAACTGAAAAAAGAGATCGAGAAAGAAGCGAAAAAATGGAAAGATCGCGCAAACGACCTGTTTGATAGCGATTTTTTTCAGGTGATCTATAGCGGTGATAACGATAAAGACGAATGGGAGAAAGAGCGCGAAGAACTGGAAGAAGAAATTAAACGTTGGGCGAAAGAAGCCAAAGAGGAACTGGATCGTATCAAAAAACGTCTGGAACAGTAA

***Protein:***

RKAEEAKKKIKRDKDEAEEKAEKLKERAREAYKKARKTEDPTEENREKWEKTVEELKKEIEKEAKKWKDRANDLFDSDFFQVIYSGDNDKDEWEKEREELEEEIKRWAKEAKEELDRIKKRLEQ

***Cys2:***

***DNA:***

AATAATTGGGAGCAGCAGAAAAAAAACATCGAGGATGATCTGGACCGTTACAAAAAACGTGCAGAAGAACTGCGTAAAGAAGCCGAAAAAGCACGCAAAGAAGCACGTAAAACCGAAGATCCGACCGAAGAGGCCAAAAAAGAATGGGAAAAACGTTGCAAAGAACTGGAAGAACGTGCCCGTAAACTGGAAGATGAAGCAAAAGATCGTGTGAACGACCTGTTTGATAGCAACTTTTTTCAGGTGATCTATAGCGGTGATAACGATGAAGAAGAGTGGAAAAAAGAAAAAGACCGTGCCGAGAAAGAAATCGAAGAATGGTTTAAACGCATCAAAGAAAAATGCGAAGAGATCAAAAAACGCCTGGAACAG

***Protein:***

NNWEQQKKNIEDDLDRYKKRAEELRKEAEKARKEARKTEDPTEEAKKEWEKR**C**KELEERARKLEDEAKDRVNDLFDSNFFQVIYSGDNDEEEWKKEKDRAEKEIEEWFKRIKEK**C**EEIKKRLEQ
