# Supplementary material for: Structure-Guided Design of a Synthetic Mimic of an Endothelial Protein C Receptor-Binding PfEMP1 Protein
Source: mSphere. 2021 Jan 6;6(1):e01081-20. doi: 10.1128/mSphere.01081-20 (PMC7845591; doi:10.1128/mSphere.01081-20)
Supplement: TABLE S2 [file mSphere.01081-20-st002.docx]

***Table S2: Data collection and refinement statistics***

|  | Cys2-EPCR |
| --- | --- |
| **Data collection** |  |
| Space group | - P3_2_21 |
| Cell dimensions |  |
| *a*, *b*, *c* (Å) | 112.73, 112.73, 168.49 |
|  α, β, γ (°) | 90.0, 90.0, 120.0 |
| Wavelength | - 1.00000 |
| Resolution (Å) | 22.38 – 3.11 (3.19 – 3.11) |
| *R*_meas_ (%) | - 7.9 (134.8) |
| *CC_1/2_* | - 1.0 (0.6) |
| Completeness (%) | - 99.5 (98.3) |
| Multiplicity | - 6.5 (6.3) |
| Number of reflections | 20508 |
|  |  |
| **Refinement** |  |
| *R*_work_ / *R*_free_ | - 20.8 / 24.7 |
| Number of residues |  |
| Protein | 437 |
| R.m.s deviations |  |
| Bond lengths (Å) | 0.01 |
| Bond angles (°) | 1.19 |
| Ramachandran plot |  |
| Favored (%) | - 96.1% |
| Allowed (%) | - 3.4% |
| Disallowed (%) | - 0.5% |
|  |  |
|  |  |

All structures were determined from one crystal.

Values in parentheses are for highest-resolution shell.
